# Muscle-building supplement use is associated with muscle dysmorphia symptomatology among Canadian adolescents and young adults

**DOI:** 10.1371/journal.pmen.0000217

**Published:** 2025-02-19

**Authors:** Kyle T. Ganson, Rachel F. Rodgers, Alexander Testa, Stuart B. Murray, Jason M. Nagata

**Affiliations:** 1 Factor-Inwentash Faculty of Social Work, University of Toronto, Toronto, Ontario, Canada; 2 Department of Applied Psychology, Northeastern University, Boston, Massachusetts, United States of America; 3 Department of Psychiatric Emergency & Acute Care, Lapeyronie Hospital, Montpellier, France; 4 Centre d’Innovation et de Recherche Clinique, Hôpital du Cotentin, Cherbourg, France; 5 Department of Management, Policy and Community Health, University of Texas Health Science Center at Houston, Houston, Texas, United States of America; 6 Department of Psychiatry and Biobehavioral Sciences, University of California, Los Angeles, Los Angeles, California, United States of America; 7 Department of Pediatrics, University of California, San Francisco, San Francisco, California, United States of America; University of Western Australia, AUSTRALIA

## Abstract

This study investigated the association between muscle-building supplement use and muscle dysmorphia symptomatology among Canadian adolescents and young adults. Data from the Canadian Study of Adolescent Health Behaviors (N = 2,731) were analyzed. Multiple linear and logistic regression analyses were used to assess the association between six commonly used muscle-building supplements (e.g., amino acids/branched-chain amino acids, creatine monohydrate, pre-workout drinks or powders, protein bars, weight/mass gainers, and whey protein shakes or powders), and an aggregated sum score of muscle-building supplements used, and muscle dysmorphia symptomatology (measured using the Muscle Dysmorphic Disorder Inventory [MDDI]), including scoring above the clinical cut-off (≥ 40 on the MDDI). The mean age of the sample was 22.9 years (*SD* = 3.9), 54.3% identified as a cisgender girl or woman, 62.4% identified as white, and 58.8% identified as heterosexual. Findings revealed that use of all six muscle-building supplements, and using a greater number of muscle-building supplements, were associated with greater total muscle dysmorphia symptomatology, as well as greater odds of meeting the clinical cut-off for muscle dysmorphia. These findings add to a growing body of literature on the association between muscle-building supplement use and muscle dysmorphia symptomatology by including multiple muscle-building supplements and utilizing a diverse, national sample of adolescents and young adults. Findings warrant further investigation and the development of intervention strategies to decrease the use of increasingly normalized muscle-building supplements and muscle dysmorphia symptomatology.

## Introduction

### Muscle-building dietary supplements

Muscle-building dietary supplements (hereafter referred to as “muscle-building supplements”) include a variety of easily accessible and legal substances that are marketed to support the development of muscles and strength, improve performance, and enhance recovery [1, 2]. Muscle-building supplements may include whey protein powders, creatine monohydrate, amino acids, and pre-workout powders or drinks. These muscle-building supplements are among the most commonly used products. For example, among samples in Canada and the United States, research has documented that 55–80% of adolescent boys and men report using whey protein powders or shakes, compared to 33–50% for adolescent girls and women [3, 4], while 14–44% of transgender and gender expansive (TGE) people report using whey protein powders or shakes [3, 5]. Similarly, over 50% of adolescent boys and men reported using creatine monohydrate, compared to only 10% of adolescent girls and women, while 5–10% of TGE people report use of creatine monohydrate [3, 5]. Moreover, research has also reported that muscle-building supplement users often consume more than one product at a time [6].

These data from Canada and the United States underscore the high prevalence of use of muscle-building supplements among the adolescent and young adult populations, highlighting the need for more research to understand the correlates of use. This continued research is particularly needed given that muscle-building supplements are loosely regulated [2] and have been reported to be associated with adverse health (e.g., disability) and social outcomes (e.g., problematic alcohol use, anabolic-androgenic steroid use, criminal behavior) [7–10]. Given the marketing framing of muscle-building supplements as supporting the increase of muscularity and strength, it is important to understand the association between muscle-building supplement use and muscle dysmorphia symptomatology, a concern characterized by the pursuit of increased muscularity.

### Muscle dysmorphia symptomatology and supplement use

Muscle dysmorphia, a specifier of body dysmorphic disorder, is characterized by the pathological pursuit of muscularity [11, 12]. The symptomatology of muscle dysmorphia often includes a variety of thoughts and feelings (e.g., drive for muscularity) and behaviors (e.g., excessive exercise, muscularity-oriented dietary practices), as well as body dissatisfaction (e.g., muscle dissatisfaction) and social and functional impairment [11–13]. Muscle dysmorphia symptomatology is more common among boys and men, compared to other genders, given the emphasis on the muscular body ideal among this population [14]. For example, among a community sample of Canadian adolescents and young adults, 26% of boys and men scored above the clinical cut-off for muscle dysmorphia using the Muscle Dysmorphic Disorder Inventory (MDDI) [15], which compares to 18% of TGE people, and 11% of girls and women [16]. Among a sample of male university students in Buenos Aires, 7% met the criteria for possible muscle dysmorphia using the Drive for Muscularity Scale [17], while the prevalence of muscle dysmorphia based on clinical interviews was 1.3% among a sample of Spanish undergraduate males [18]. Prior research has also documented that 2.2% of adolescent boys (compared to 1.4% of adolescent girls) in a nationally representative sample of high school students in Australia met clinical criteria for muscle dysmorphia [19]. Finally, in samples in Italy and the United States, transgender men often displayed equivalent, and at times higher, muscle dysmorphia symptoms compared to cisgender men, transgender women, and gender expansive people [5, 20].

To date, few empirical studies have investigated the association between muscle-building supplement use and muscle dysmorphia symptomatology using epidemiological data. Much of the prior research has been either among specific samples, such as those with a diagnosis of muscle dysmorphia [13], or has solely investigated the association between anabolic-androgenic steroid use and muscle dysmorphia symptomatology [16, 21]. This prior research has also reported that individuals who experience muscle dysmorphia symptomatology often consume multiple muscle-building supplements [13, 22]. Consistent with this, other research has documented that muscle-building supplement use is more common among adolescent and young adult males who report muscularity concerns [23–25]. Similarly, the use of appearance- and performance-enhancing drugs and substances, including both dietary supplement use and anabolic-androgenic steroid use, is associated with muscle dysmorphia symptomatology among transgender men and gender expansive people [5] and sexual minority adults [26].

Overall, the prior research on the association between muscle-building supplement use and muscle dysmorphia symptomatology presents important limitations. These include the samples in prior research being limited to specific groups (e.g., bodybuilders) and a lack of gender diversity. Additionally, prior research has conflated multiple muscle-building supplements and illicit drugs, such as anabolic-androgenic steroids, into a single group. Furthermore, prior research has not specifically investigated how individual muscle-building supplements, and the number of muscle-building supplements used, may be associated with muscle dysmorphia symptomatology. Therefore, the aim of this study was to determine the association between muscle-building supplement use and muscle dysmorphia symptomatology among a community sample of adolescents and young adults from all 13 provinces and territories in Canada. Overall, it was hypothesized that significant associations between the use of muscle-building supplements and greater muscle dysmorphia symptomatology would be found, including with scoring above the clinical cut-off (score of ≥ 40) for muscle dysmorphia on the MDDI. It was also hypothesized that a greater number of muscle-building supplements used would be associated with greater muscle dysmorphia symptomatology, including scoring above the clinical cut-off.

## Methods

Data from 2,731 participants in the Canadian Study of Adolescent Health Behaviors, a nationwide study of adolescents and young adults ages 16–30 years across Canada, were analyzed. Participants were recruited starting November 1, 2021 and ending December 31, 2021 via Instagram and Snapchat advertisements without specific targeting criteria, except for age and location. The data were collected online via Qualtrics, and participants were able to enter a raffle for one of three Apple iPads for their participation. To prevent survey infiltration by bots, several recommended techniques were added to the survey, including reCAPTCHA verification and attention checks [27]. Additionally, several Qualtrics features were utilized, including “prevent multiple submissions” (i.e., preventing participants from taking the survey multiple times) and “block indexing options” (i.e., blocking search engines from including the survey in their search results).

### Ethics statement

Ethics approval was obtained from the Health Sciences Research Ethics Board (REB) at the University of Toronto (#41707) and written informed consent (via checkbox) was obtained from all participants (consent from guardians was not required from those under 18 years as approved by the REB).

### Measures

Use of muscle-building dietary supplements in the past 12 months was assessed based on the question, “Over the past 12 months, have you used any of the following appearance and performance-enhancing dietary supplements and substances?” Participants were then provided with a list of legal dietary supplements to select from, including amino acids/branched-chain amino acids (BCAAs), creatine monohydrate, pre-workout drinks or powders, protein bars, weight/mass gainers, and whey protein shakes or powders. The muscle-building dietary supplements were listed individually and independently for participants to select. These muscle-building dietary supplements were chosen given the overall high prevalence of use among adolescents and young adults [3, 4], as well as the ease of accessibility of these products in Canada given their legal status [2, 28]. An aggregated sum score of muscle-building supplements used was created by adding all the muscle-building dietary supplements (range: 0–6).

Muscle dysmorphia symptomatology was assessed based on the Muscle Dysmorphic Disorder Inventory (MDDI) [15], the most widely used measure of muscle dysmorphia symptomatology [12]. The MDDI has 13 items and uses a five-point Likert-type scale (1 = *never*; 5 = *always*). The total score is calculated by summing the 13 scale items, with a higher score indicating greater symptom severity of muscle dysmorphia. The MDDI is also comprised of three subscales, including Drive for Size (e.g., “I wish I could get bigger.”), Appearance Intolerance (e.g., “I feel like I have too much body fat.”), and Functional Impairment (e.g., “I feel depressed when I miss one or more workout days.”). Internal reliability using Cronbach’s alpha was good for the MDDI total score (α = 0.80), Drive for Size subscale (α = 0.87), Appearance Intolerance subscale (α = 0.84), and Functional Impairment subscale (α = 0.85). Note that scores on the MDDI were comparable to prior research among community samples [15, 29, 30]. A clinical cut-off score of 40 or higher on the MDDI total score was utilized to identify individuals at clinical risk for muscle dysmorphia [16, 29, 31, 32].

Sociodemographic variables included self-reported age, race/ethnicity, sexual orientation, and highest completed education. Gender included those who identified as cisgender girls and women, cisgender boys and men, and transgender and gender expansive participants.

### Statistical analysis

Descriptive statistics were used to characterize the sample. Multiple linear regression analyses were conducted with each individual muscle-building supplement, and the sum score, as the independent variables (all analyzed separately), and muscle dysmorphia symptomatology, including the three subscales and total score, as the dependent variables (all analyzed separately). A total of 28 linear regressions were conducted. Multiple logistic regression analyses were also conducted with each individual muscle-building supplement, and the sum score, as the independent variables (all analyzed separately), and the clinical cut-off for muscle dysmorphia as the dependent variable, for a total of 7 logistic regressions. All regression analyses adjusted for the sociodemographic variables (age, gender, race/ethnicity, sexual orientation, highest completed education) given prior research on muscle-building supplement use and muscle dysmorphia [3, 16, 26]. Supplementary analyses were conducted stratifying all models by gender (except for TGE participants due to small cell sizes). Listwise deletion was used to account for missing data given the large sample raising minimal issues with statistical power [33, 34]. To account for potential Type I error, the Benjamini-Hochberg procedure with a 20% false-discovery rate was used to determine statistical significance [35]. Statistical analyses were conducted using StataMP 18.0.

## Results

The sample was demographically diverse, with a mean age of 22.9 years (*SD* = 3.9), 54.3% identifying as girls or women, 62.4% white, and 58.8% heterosexual (Table 1). Protein bars (63.4%) were the most commonly used muscle-building supplement among those assessed, followed by whey protein shakes or powders (63.1%) and creatine monohydrate (25.5%). The mean number of muscle-building supplements used was 2.0 (*SD* = 1.5) and 79.6% of participants reported using at least one muscle-building supplement in the past 12 months. The mean total score on the MDDI was 31.7 (*SD* = 8.1), and mean scores for the subscales were 10.9 (*SD* = 5.2) for Drive for Size, 8.9 (*SD* = 4.0) for Functional Impairment, and 11.8 (*SD* = 4.3) for Appearance Intolerance. Finally, 18.0% of the sample scored 40 or higher on the MDDI, representing the clinical cut-off for muscle dysmorphia. See S1 Table for descriptive statistics by gender identity. Note that the use of all muscle-building supplements was significantly higher among boys and men compared to girls, women, and TGE participants. A similar pattern was found for MDDI scores, with the exception of Appearance Intolerance scores being higher among TGE participants and girls and women compared to boys and men. Additionally, 11.8% of cisgender girls and women, 26.2% of cisgender boys and men, and 19.3% of transgender and gender expansive participants scored above the clinical cut-off (≥ 40 on MDDI).

**Table 1:** Sample characteristics of participants in the Canadian Study of Adolescent Health Behaviors (*N* = 2,731).

	%
Age (*M* [*SD*])	22.92 (3.90)
Gender	
Girls and Women	54.32
Boys and Men	39.02
Transgender/Gender Expansive	6.66
Race/ethnicity	
White	62.45
Black	3.15
Asian	16.94
Other	7.30
Multi-Racial	10.16
Sexual Orientation	
Heterosexual	58.83
Gay/Lesbian	8.04
Bisexual	18.02
Queer, Questioning, Other	15.12
Highest Completed Education	
High School Diploma or Less	44.11
College or Undergraduate Degree	42.79
Master’s Degree or Higher	11.71
Other	1.39
Muscle-Building Dietary Supplements Use (Yes Responses), Past 12 Months	
Amino Acids/BCAAs	21.27
Creatine Monohydrate	25.52
Pre-Workout Drinks or Powders	24.50
Protein Bars	63.42
Weight/Mass Gainers	4.54
Whey Protein Shakes or Powders	63.09
Sum Score (Range 0–6; *M* [*SD*])	2.03 (1.54)
Muscle Dysmorphic Disorder Inventory	
Drive for Size (*M* [*SD*])	10.91 (5.25)
Functional Impairment (*M* [*SD*])	8.94 (4.02)
Appearance Intolerance (*M* [*SD*])	11.84 (4.34)
Total Score (*M* [*SD*])	31.72 (8.08)
Clinical Cut-Off (≥ 40 on MDDI)	18.02

*M* = Mean; *SD* = Standard deviation; BCAA = Branched-chain amino acids; MDDI = Muscle Dysmorphic Disorder Inventory

**Fig 1:**
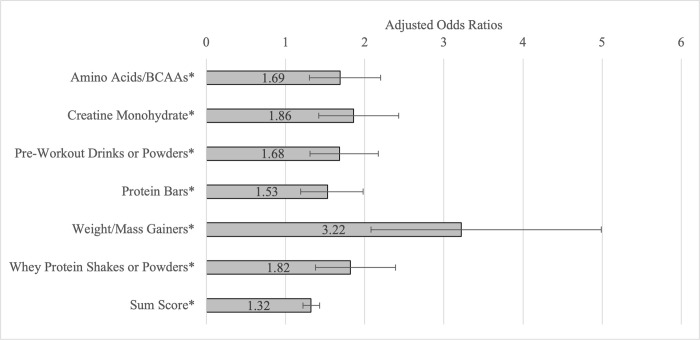
Odds of meeting muscle dysmorphia clinical cut-off (≥ 40 on MDDI) by use of muscle-building supplements. Note: Each bar represents the abbreviated output of a separate logistic regression analysis (7 separate regression analyses in total) with each muscle-building supplement as the independent variables and muscle dysmorphia clinical cut-off as the dependent variable. Each muscle-building supplement was analyzed separately and controlled for age, gender, race/ethnicity, sexual orientation, and highest completed education. Error bars represent 95% confidence intervals. Error bars represent 95% confidence intervals. MDDI = Muscle Dysmorphic Disorder Inventory. * Indicates statistical significance using the Benjamini-Hochberg procedure with a 20% false-discovery rate.

As predicted, significant associations were present between muscle-building supplement use and greater muscle dysmorphia symptomatology among the sample (Table 2). Use of each muscle-building supplement in the past 12 months was associated with greater total muscle dysmorphia symptomatology, Drive for Size symptomatology, and Functional Impairment symptomatology. Use of amino acids/BCAAs, creatine monohydrate, pre-workout drinks or powders, weight/mass gainers, and whey protein shakes or powder in the past 12 months was associated with lower Appearance Intolerance symptomatology. Finally, a greater number of muscle-building supplements used in the past 12 months was associated with greater total muscle dysmorphia symptomatology, Drive for Size symptomatology, Functional Impairment symptomatology, and lower Appearance Intolerance symptomatology. Use of each muscle-building supplement in the past 12 months was associated with greater odds of reporting a score above the clinical cut-off (score of ≥ 40 on the MDDI) for muscle dysmorphia (Fig 1).

**Table 2:** Associations between muscle-building dietary supplement use in the past 12 months and muscle dysmorphia symptomatology.

	Drive for Size		Functional Impairment		Appearance Intolerance		MDDI Total Score	
Muscle-Building Dietary Supplements Use, Past 12 Months	*B* (95% CI)^a^	*p*	*B* (95% CI)^a^	*p*	*B* (95% CI)^a^	*p*	*B* (95% CI)^a^	*p*
Amino Acids/BCAAs	**1.08 (0.64, 1.52)**	**< .001**	**1.81 (1.41, 2.23)**	**< .001**	**-0.70 (-1.11, -0.29)**	**.001**	**2.20 (1.38, 3.01)**	**< .001**
Creatine Monohydrate	**1.98 (1.52, 2.43)**	**< .001**	**2.01 (1.59, 2.44)**	**< .001**	**-0.91 (-1.34, -0.49)**	**< .001**	**3.01 (2.17, 3.85)**	**< .001**
Pre-Workout Drinks or Powders	**1.44 (1.02, 1.85)**	**< .001**	**1.84 (1.45, 2.22)**	**< .001**	**-0.47 (-0.86, -0.08)**	**.018**	**2.82 (2.05, 5.59)**	**< .001**
Protein Bars	**0.90 (0.53, 1.27)**	**< .001**	**1.35 (1.01, 1.69)**	**< .001**	-0.24 (-0.58, 0.10)	.166	**2.00 (1.33, 2.68)**	**< .001**
Weight/Mass Gainers	**4.74 (3.89, 5.59)**	**< .001**	**1.88 (1.06, 7.71)**	**< .001**	**-0.91 (-1.71, 0.10)**	**.028**	**5.71 (4.12, 7.30)**	**< .001**
Whey Protein Shakes or Powders	**1.29 (0.91, 1.67)**	**< .001**	**1.83 (1.47, 2.18)**	**< .001**	**-0.81 (-1.17, -0.46)**	**< .001**	**2.28 (1.57, 2.99)**	**< .001**
Sum Score (Range 0–6)	**0.70 (0.58, 0.72)**	**< .001**	**0.85 (0.74, 0.96)**	**< .001**	**-0.30 (-0.41, -0.18)**	**< .001**	**1.24 (1.02, 1.47)**	**< .001**

Note: Each cell represents the abbreviated outputs of 28 unstandardized linear regression models with each muscle-building supplement (analyzed separately) as the independent variable and muscle dysmorphia symptomatology (each subscale and total score, analyzed separately) as the dependent variable.

**Boldface** indicates statistical significance using the Benjamini-Hochberg procedure with a 20% false-discovery rate.

^a^ Adjusted for age, gender, race/ethnicity, sexual orientation, and highest completed education.

MDDI = Muscle Dysmorphic Disorder Inventory; CI = Confidence interval

Gender-stratified models for all analyses are displayed in S2 and S3 Tables. Overall, similar patterns of association between girls and women and boys and men emerged. However, several key differences were present that primarily focused on the association between the use of muscle-building supplements and Appearance Intolerance. For example, among boys and men, but not girls and women, a significant association was found between the use of weight/mass gainers in the past 12 months and lower Appearance Intolerance scores. Additionally, for girls and women, but not boys and men, a significant association was found between the use of amino acids/BCAAs in the past 12 months and lower Appearance Intolerance scores. Lastly, while the use of all six muscle-building supplements in the past 12 months was significantly associated with greater odds of reporting a score above the clinical cut-off for muscle dysmorphia for girls and women, only the use of creatine monohydrate and weight/mass gainers in the past 12 months were associated among boys and men.

## Discussion

The findings from this study underscore the association between the use of muscle-building supplements and greater muscle dysmorphia symptomatology among a national, community sample of adolescents and young adults in Canada. Additionally, the use of muscle-building supplements was associated with greater odds of above the clinical cut-off for muscle dysmorphia. Notably, the use of weight/mass gainers and creatine monohydrate had the strongest effect sizes across all analyses. Furthermore, findings underscore the strong association between the use of multiple muscle-building supplements and muscle dysmorphia symptomatology. These findings support the hypotheses of this study and align with prior research [13, 22]. However, this study adds to the prior research, particularly from the United States, by investigating specific muscle-building supplements and utilizing a diverse, non-clinical sample of adolescents and young adults. Additionally, the study examines the Canadian context, which generally perpetuates notions of the muscular, lean, and fit body ideal, and overall healthism, that is pervasive within North American and Western culture [36, 37]. Finally, the study explores the association between muscle-building supplement use and symptom clusters of muscle dysmorphia.

First, it is important to note that all muscle-building supplements were associated with greater total muscle dysmorphia symptomatology, as well as greater Drive for Size and Functional Impairment symptoms. Regarding Drive for Size, muscle-building supplements have purported benefits of supporting muscle growth and development [1], which would align with higher scores of Drive for Size. Indeed, prior research has documented that the use of muscle-building supplements is more common among adolescent males who report drive for muscularity [25]. The use of weight/mass gainers had the strongest effect size for Drive for Size, likely given that these high-calorie products are intended to support the muscular growth of users. It has also been reported that using whey protein [38, 39], creatine monohydrate [40], and pre-workout [41], for example, can have positive ergogenic benefits when coupled with weight training, underscoring their strong association with Drive for Size.

A key feature of muscle dysmorphia includes social and functional impairment, which may include prioritizing weight training and muscularity-oriented practices above social, occupational, and educational responsibilities [11, 13]. The findings from this study may imply that the use of muscle-building supplements is a contributing factor to the functional capacity of individuals who experience muscle dysmorphia symptomatology. Prior research among people diagnosed with muscle dysmorphia found that these individuals reported significant distress and loss of control when not meeting daily macronutrient goals, which was a precursor for muscle-building supplement use [13]. Interestingly, creatine monohydrate had the strongest effect size with Functional Impairment. Creatine appears to have the most evidence supporting muscle growth and strength development, when consumed in conjunction with weight training [42]; therefore, users may be particularly aware of the importance of consuming this muscle-budling supplement to support their goals and fear not consuming it based on their perceived macronutritional and exercise needs.

The use of muscle-building supplements was associated with lower Appearance Intolerance. This finding contradicts prior research that has shown that adolescent and young adult males who report muscularity concerns commonly use muscle-building supplements [23]. However, with the Appearance Intolerance subscale measuring intolerance of one’s current appearance, it may be that the use of muscle-building supplements supports the goals of users, namely, to increase muscle mass and strength primarily through weight training, which may decrease their muscle dissatisfaction and avoidance of showing their body in social situations (i.e., Appearance Intolerance). Specifically, weight/mass gainers and creatine monohydrate had the strongest associations with lower Appearance Intolerance scores, which, as noted, may be due to the actual or perceived effectiveness of these supplements towards meeting one’s body image goals (i.e., muscularity). Indeed, prior research has reported that muscle-building supplement users, when coupled with weight training, often experience positive body image effects [43]. Additionally, research has documented that muscle-building supplements, such as whey protein, creatine monohydrate, and pre-workout, can have positive ergogenic effects [39, 41, 44].

It should be noted that patterns of differences emerged in the association between muscle-building supplement use and muscle dysmorphia symptomatology between boys and men and girls and women. Namely, more significant associations were found between muscle-building supplement use and muscle dysmorphia symptomatology among girls and women. For example, all six supplements, and a greater number of muscle-building supplements used, were all associated with greater muscle dysmorphia symptomatology and scoring above the clinical cut-off, all with relatively strong effect sizes. This counters that of the findings among boys and men, where, for example, only the use of creatine monohydrate, weight/mass gainers, and a greater number of muscle-building supplements used, were associated with scoring above the clinical cut-off for muscle dysmorphia. These differing findings may be due to muscle-building supplement use being more ubiquitous among boys and men [3, 4], meaning that use may occur among boys and men independent from muscle dysmorphia symptomatology. Therefore, muscle-building supplement use may be considered a stronger correlate of muscle dysmorphia symptomatology among girls and women. This also may be related to body composition, whereby males typically have more muscle mass than females [45]. Therefore, females with high muscle dissatisfaction and drive for muscularity may use muscle-building supplements with the intention of addressing this body compositional difference, which may in turn perpetuate symptoms of muscle dysmorphia. Indeed, future research, including qualitative methods, is needed to explore the mechanisms underpinning these unique findings. Prevention efforts should consider the unique use of muscle-building supplements among girls and women, and assessment for muscle dysmorphia symptomatology among girls and women who use muscle-building supplements may be warranted.

The findings from this study have important implications for health and mental health care professionals and policymakers. Health and mental health care professionals should consider assessing for muscle-building supplements and muscle dysmorphia symptomatology among adolescent and young adult clients [46]. Given the common use of muscle-building supplements among these populations, assessment of use may be included as part of routine practice. Specifically, assessment should include identifying the specific supplements used, the number of supplements used, and frequency and dose of use, as well as the purpose of use [46]. Such information will provide greater context for prevention and intervention efforts. Providing education on the potential benefits and risks associated with muscle-building supplement use is also warranted [47], including information on muscle dysmorphia symptomatology. Additionally, assessing the number of muscle-building supplements used is warranted as individuals often use multiple supplements [13, 43] and, as evidenced by this study, is strongly associated with muscle dysmorphia symptomatology. Policymakers may consider strengthening the regulations of natural health products (i.e., muscle-building supplements) in Canada to protect the health and well-being of users [2], particularly given that many muscle-building supplement users are adolescents and may not have the health literacy skills to make informed health behavior decisions [48]. This may include deterring use, increasing oversight of manufacturers and advertising, and providing more public awareness of the benefits and risks associated with use. Additionally, greater regulations for advertisements and content, particularly on social media [49], may reduce misinformation on muscle-building supplements and uninformed use among young people. Finally, it is important for future research to investigate the association between muscle-building dietary supplement use and muscle dysmorphia symptomatology among athletes, as this group may be at particularly high risk.

Despite the important findings and implications of this study, limitations should also be noted and addressed in future investigations. The data were collected via non-probability sampling methods, which decreases the external validity of the findings. However, the sample was demographically diverse and included participants from all 13 provinces and territories in Canada. Additionally, the data are comparable with Canadian breakdowns of race/ethnicity among adolescents and young adults (i.e., 36.3% visible minority in the current study versus 27.0% based on the 2016 Canadian census) [50]. The data were cross-sectional, and thus the findings should not be interpreted as causal. Similarly, there may be bidirectional relationships between muscle-building supplement use and muscle dysmorphia symptomology. Future research is needed in a national, longitudinal cohort study to replicate the findings and assess whether muscle-building supplement use is associated with muscle dysmorphia symptomatology over time. The study was limited to investigating any or no use of muscle-building supplements, as well as past 12-month use, which may introduce recall bias. There was also no method of verifying participants’ responses to the muscle-building dietary supplements item. Future research should investigate how the frequency and dose of use of muscle-budling supplements are associated with muscle dysmorphia symptomatology. However, the study did include a sum score of muscle-building supplements, which provides important evidence of the accumulated effect of use on muscle dysmorphia symptomatology. Finally, the study was not able to explicate specific mechanisms that link muscle-building supplement use with muscle dysmorphia symptomatology, which is an important area of future investigation. Strengths of the study include the large, national, and diverse sample of adolescents and young adults across Canada, as well as the use of multiple individual muscle-building supplements.

## Conclusion

Findings from this study underscore that muscle-building supplement use was associated with greater overall muscle dysmorphia symptomatology, including scoring above the clinical cut-off, as well as symptoms related to Drive for Size and Functional Impairment. Conversely, muscle-building supplement use was associated with fewer symptoms of Appearance Intolerance (i.e., muscle dissatisfaction). These findings expand prior research by investigating these associations among an epidemiological sample of adolescents and young adults across Canada and investigating a number of commonly used muscle-building supplements. Health and mental health care professionals should be alerted to these findings, given the regular use of muscle-building supplements and the ubiquity of the muscular, lean, and toned body ideal. Future research is needed to identify the directionality and mechanisms underlying these associations.

## Supporting information

S1 Table Sample characteristics of participants in the Canadian study of adolescent health behaviors by gender (*N* = 2,731). (DOCX)

S2 Table Associations between muscle-building dietary supplement use in the past 12 months and muscle dysmorphia symptomatology, stratified by gender. (DOCX)

S3 Table Associations between muscle-building supplement use and muscle dysmorphia clinical cut-off (≥ 40 on MDDI), stratified by gender. (DOCX)
