# Supplementary material for: Muscle-building supplement use is associated with muscle dysmorphia symptomatology among Canadian adolescents and young adults
Source: PLOS Ment Health. 2025 Feb 19;2(2):e0000217. doi: 10.1371/journal.pmen.0000217 (PMC12798181; doi:10.1371/journal.pmen.0000217)
Supplement: S1 Table — (DOCX) [file pmen.0000217.s001.docx]

| S1 Table.  Sample Characteristics of Participants in the Canadian Study of Adolescent Health Behaviors by Gender (*N* = 2,731) | | | | |
| --- | --- | --- | --- | --- |
|  | Cisgender Girls and Women | Cisgender Boys and Men | Transgender and Gender Expansive Individuals |  |
|  | % | % |  | *p*^a^ |
| Age (*M* [*SD*]) | 23.1 (3.9) | 22.8 (3.9) | 21.8 (3.9) | .001 |
| Race/ethnicity |  |  |  | < .001 |
| White | 65.2 | 57.5 | 70.6 |  |
| Black | 3.2 | 3.1 | 2.8 |  |
| Asian | 14.6 | 21.0 | 12.4 |  |
| Other | 6.6 | 9.1 | 2.8 |  |
| Multi-Racial | 10.4 | 9.3 | 11.3 |  |
| Sexual Orientation |  |  |  | < .001 |
| Heterosexual | 57.3 | 70.2 | 5.6 |  |
| Gay/Lesbian | 2.4 | 14.4 | 16.4 |  |
| Bisexual | 23.8 | 8.7 | 24.9 |  |
| Queer, Questioning, Other | 16.5 | 6.7 | 53.1 |  |
| Highest Completed Education |  |  |  | .004 |
| High School Diploma or Less | 41.7 | 45.1 | 57.1 |  |
| College or Undergraduate Degree | 43.8 | 43.2 | 33.3 |  |
| Master’s Degree or Higher | 12.9 | 10.7 | 7.9 |  |
| Other | 1.6 | 1.0 | 1.7 |  |
| Muscle-Building Dietary Supplements Use (Yes Responses), Past 12 Months |  |  |  |  |
| Amino Acids/BCAAs | 15.0 | 32.2 | 8.8 | < .001 |
| Creatine Monohydrate | 9.7 | 50.3 | 9.9 | < .001 |
| Pre-Workout Drinks or Powders | 18.4 | 35.7 | 9.4 | < .001 |
| Protein Bars | 59.2 | 70.9 | 55.2 | < .001 |
| Weight/Mass Gainers | 1.4 | 9.1 | 3.3 | < .001 |
| Whey Protein Shakes or Powders | 51.9 | 82.5 | 41.4 | < .001 |
| Sum Score (Range 0-6; *M* [*SD*]) | 1.6 (1.4) | 2.8 (1.5) | 1.3 (1.3) | < .001 |
| Muscle Dysmorphic Disorder Inventory |  |  |  |  |
| Drive for Size (*M* [*SD*]) | 8.3 (3.8) | 14.7 (4.8) | 9.5 (4.6) | < .001 |
| Functional Impairment (*M* [*SD*]) | 8.7 (4.1) | 9.4 (3.8) | 7.7 (4.1) | < .001 |
| Appearance Intolerance (*M* [*SD*]) | 13.0 (4.0) | 9.7 (4.0) | 14.2 (4.2) | < .001 |
| Total Score (*M* [*SD*]) | 30.1 (7.3) | 33.9 (8.6) | 31.5 (7.6) | < .001 |
| Clinical Cut-Off (≥ 40 on MDDI) | 11.8 | 26.2 | 19.3 | < .001 |
| ^a^ Statistical significance determined using chi-square tests for categorical variables and one-way ANOVAs for continuous variables.  *p* < .05 indicates statistical significance.  *M* = Mean; *SD* = Standard deviation; BCAA = Branched-chain amino acids; MDDI = Muscle Dysmorphic Disorder Inventory | | | | |
