# Supplementary material for: Muscle-building supplement use is associated with muscle dysmorphia symptomatology among Canadian adolescents and young adults
Source: PLOS Ment Health. 2025 Feb 19;2(2):e0000217. doi: 10.1371/journal.pmen.0000217 (PMC12798181; doi:10.1371/journal.pmen.0000217)
Supplement: S2 Table — (DOCX) [file pmen.0000217.s002.docx]

| S2 Table.  Associations between Muscle-Building Dietary Supplement Use in the Past 12 Months and Muscle Dysmorphia Symptomatology, Stratified by Gender | | | | | | | | |
| --- | --- | --- | --- | --- | --- | --- | --- | --- |
| Panel A. Cisgender Girls and Women | | | | | | | | |
|  | Drive for Size | | Functional Impairment | | Appearance Intolerance | | MDDI Total Score | |
| Muscle-Building Dietary Supplements Use, Past 12 Months | *B* (95% CI)^a^ | *p* | *B* (95% CI)^a^ | *p* | *B* (95% CI)^a^ | *p* | *B* (95% CI)^a^ | *p* |
| Amino Acids/BCAAs | **1.73 (1.14, 2.32)** | **< .001** | **2.49 (1.87, 3.11)** | **< .001** | **-0.91 (-1.53. -0.30)** | **.004** | **3.31 (2.19, 4.43)** | **< .001** |
| Creatine Monohydrate | **3.22 (2.50, 3.94)** | **< .001** | **3.17 (2.40, 3.94)** | **< .001** | **-1.33 (-2.09, -0.56)** | **.001** | **4.99 (3.61, 6.37)** | **< .001** |
| Pre-Workout Drinks or Powders | **1.65 (1.11, 2.19)** | **< .001** | **2.06 (2.03, 3.18)** | **< .001** | -0.38 (-0.94, 0.20) | .209 | **3.87 (2.84, 4.90)** | **< .001** |
| Protein Bars | **0.79 (0.36, 1.22)** | **< .001** | **1.59 (1.13, 2.05)** | **< .001** | -0.08 (-0.52, 0.36) | .716 | **2.33 (1.51, 3.15)** | **< .001** |
| Weight/Mass Gainers | **7.73 (6.00, 9.47)** | **< .001** | **2.89 (0.99, 4.80)** | **.003** | -0.28 (-2.14, 1.57) | .767 | **10.32 (6.97, 13.67)** | **< .001** |
| Whey Protein Shakes or Powders | **1.33 (0.91, 1.75)** | **< .001** | **2.07 (1.62, 2.51)** | **< .001** | **-0.66 (-1.10, -0.21)** | **.004** | **2.70 (1.90, 3.50)** | **< .001** |
| Sum Score (Range 0-6) | **0.74 (0.59, 0.90)** | **< .001** | **1.05 (0.89, 1.20)** | **< .001** | **-0.25 (-0.42, 0.09)** | **.002** | **1.53 (1.24, 1.81)** | **< .001** |
| Panel B. Cisgender Boys and Men | | | | | | | | |
|  | Drive for Size | | Functional Impairment | | Appearance Intolerance | | MDDI Total Score | |
| Muscle-Building Dietary Supplements Use, Past 12 Months | *B* (95% CI)^a^ | *p* | *B* (95% CI)^a^ | *p* | *B* (95% CI)^a^ | *p* | *B* (95% CI)^a^ | *p* |
| Amino Acids/BCAAs | 0.39 (-0.30, 1.08) | 0.270 | **1.15 (0.59, 1.70)** | **< .001** | -0.47 (-1.02, 0.09) | .099 | 1.08 (-0.16, 2.33) | .089 |
| Creatine Monohydrate | **1.22 (0.58, 1.86)** | **< .001** | **1.43 (0.92, 1.94)** | **< .001** | **-0.67 (-1.18, -0.16)** | **.011** | **1.93 (0.76, 3.09)** | **.001** |
| Pre-Workout Drinks or Powders | **1.09 (0.42, 1.76)** | **.001** | **1.12 (0.58, 1.66)** | **< .001** | -0.44 (-0.97, 0.09) | .107 | **1.82 (0.61, 3.03)** | **.003** |
| Protein Bars | **0.91 (0.20, 1.62)** | **.012** | **0.92 (0.35, 1.50)** | **.002** | **-0.46 (-1.03, 0.10)** | .112 | **1.30 (0.01, 2.59)** | **.049** |
| Weight/Mass Gainers | **3.54 (2.44, 4.64)** | **< .001** | **1.53 (0.62, 2.45)** | **.001** | **-0.98 (-1.88, -0.08)** | **.032** | **4.05 (2.02, 6.08)** | **< .001** |
| Whey Protein Shakes or Powders | **1.00 (0.14, 1.86)** | **.023** | **1.26 (0.57, 1.95)** | **< .001** | **-1.13 (-1.81, -0.45)** | **.001** | 1.12 (-0.43, 2.68) | .157 |
| Sum Score (Range 0-6) | **0.57 (0.36, 0.79)** | **< .001** | **0.62 (0.45, 0.79)** | **< .001** | **-0.32 (-0.49, -0.15)** | **< .001** | **0.86 (0.47, 1.25)** | **< .001** |
| Note: Each cell represents the abbreviated outputs of 28 unstandardized linear regression models with each muscle-building supplement (analyzed separately) as the independent variable and muscle dysmorphia symptomatology (each subscale and total score, analyzed separately) as the dependent variable.  **Boldface** indicates statistical significance using the Benjamini-Hochberg procedure with a 20% false-discovery rate.  ^a^ Adjusted for age, race/ethnicity, sexual orientation, and highest completed education.  MDDI = Muscle Dysmorphic Disorder Inventory; CI = Confidence interval | | | | | | | | |
