# Supplementary material for: Muscle-building supplement use is associated with muscle dysmorphia symptomatology among Canadian adolescents and young adults
Source: PLOS Ment Health. 2025 Feb 19;2(2):e0000217. doi: 10.1371/journal.pmen.0000217 (PMC12798181; doi:10.1371/journal.pmen.0000217)
Supplement: S3 Table — (DOCX) [file pmen.0000217.s003.docx]

| S3 Table.  Associations between Muscle-Building Supplement Use and Muscle Dysmorphia Clinical Cut-Off (≥ 40 on MDDI), Stratified by Gender | | |
| --- | --- | --- |
|  | Panel A. Cisgender Girls and Women | |
| Muscle-Building Dietary Supplements Use, Past 12 Months | AOR (95% CI)^a^ | *p* |
| Amino Acids/BCAAs | **2.88 (1.89-4.39** | **< .001** |
| Creatine Monohydrate | **3.29 (2.03-5.30)** | **< .001** |
| Pre-Workout Drinks or Powders | **2.14 (1.61-3.60)** | **< .001** |
| Protein Bars | **1.78 (1.21-2.63)** | **.004** |
| Weight/Mass Gainers | **9.69 (3.62-25.97)** | **< .001** |
| Whey Protein Shakes or Powders | **2.34 (1.60-3.43)** | **< .001** |
| Sum Score (Range 0-6) | **1.53 (1.35-1.73)** | **< .001** |
|  | Panel B. Cisgender Boys and Men | |
| Muscle-Building Dietary Supplements Use, Past 12 Months | AOR (95% CI)^a^ | *p* |
| Amino Acids/BCAAs | 1.24 (0.88-1.73) | .219 |
| Creatine Monohydrate | **1.43 (1.03-1.95)** | **.034** |
| Pre-Workout Drinks or Powders | 1.37 (0.99-1.91) | .057 |
| Protein Bars | 1.34 (0.93-1.93) | .111 |
| Weight/Mass Gainers | **2.22 (1.34-3.68)** | **.002** |
| Whey Protein Shakes or Powders | 1.22 (0.79-1.88) | .356 |
| Sum Score (Range 0-6) | **1.18 (1.06-1.32)** | **.003** |
| Note: Each cell represents the abbreviated outputs of 7 logistic regression analyses with each muscle-building supplement (analyzed separately) as the independent variables and muscle dysmorphia clinical cut-off as the dependent variable.  **Boldface** indicates statistical significance using the Benjamini-Hochberg procedure with a 20% false-discovery rate.  ^a^ Adjusted for age, race/ethnicity, sexual orientation, and highest completed education.  MDDI = Muscle Dysmorphic Disorder Inventory; CI = Confidence interval | | |
